# Current status and perspectives in immunotherapy for metastatic melanoma

**DOI:** 10.18632/oncotarget.23746

**Published:** 2018-01-03

**Authors:** Riccardo Marconcini, Francesco Spagnolo, Luigia Stefania Stucci, Simone Ribero, Elena Marra, Francesco De Rosa, Virginia Picasso, Lorenza Di Guardo, Carolina Cimminiello, Stefano Cavalieri, Laura Orgiano, Enrica Tanda, Laura Spano, Alfredo Falcone, Paola Queirolo, for the Italian Melanoma Intergroup (IMI)

**Affiliations:** ^1^ Unit of Medical Oncology 2, Azienda Ospedaliera-Universitaria Pisana, Department of Translational Research and New Technologies in Medicine and Surgery, University of Pisa, Italy; ^2^ Department of Medical Oncology, IRCCS AOU San Martino-Istituto Nazionale per la Ricerca sul Cancro, Genova, Italy; ^3^ Medical Oncology Unit, Department of Biomedical Sciences and Clinical Oncology, University of Bari, Bari, Italy; ^4^ Dermatologic Clinic, Department of Medical Sciences, University of Turin, Turin, Italy; ^5^ Istituto Scientifico Romagnolo per lo Studio e la Cura dei Tumori, IRST IRCCS, Meldola, Italy; ^6^ Fondazione IRCCS Istituto Nazionale dei Tumori, Milan, Italy; ^7^ AOU Cagliari, Department of Medical Oncology, University of Cagliari, Cagliari, Italy

**Keywords:** melanoma, immunotherapy, ipilimumab, pembrolizumab, nivolumab

## Abstract

Metastatic melanoma was the first malignancy in which immune checkpoint inhibitors demonstrated their successful efficacy. Currently, the knowledge on the interaction between the immune system and malignant disease is steadily increasing and new drugs and therapeutic strategies are overlooking in the clinical scenario. To provide a comprehensive overview of immune modulating drugs currently available in the treatment of melanoma as well as to discuss of possible future strategies in the metastatic melanoma setting, the present review aims at analyzing controversial aspects about the optimal immunomodulating treatment sequences, the search for biomarkers of efficacy of immunocheckpoint inhibitors, and innovative combinations of drugs currently under investigation.

## INTRODUCTION

In the last ten years, it has been amply demonstrated how the immune system represents a safe therapeutic target for solid tumors of different origin, first of all melanoma, characterized by a strong immunogenicity. Through the control and enhancement of the main immune checkpoints, therefore, we could stimulate the immune system to its fullest potential, ensuring control of the disease, and in a significant percentage of cases, response to treatment.

Monoclonal antibodies (mAbs) able to block the immune checkpoints cytotoxic T-lymphocyte antigen-4 (CTLA-4), programmed cell death protein 1 (PD-1) and its ligand (PD-L) have demonstrated high activity in metastatic melanoma and other solid tumors.

CTLA-4 is an inhibitory molecule present on T cells: during the interaction between antigen presenting cells and lymphocytes, CTLA4 competes with co-stimulatory signals and interrupts T cell priming. By blocking CTLA-4, the inhibitory effect on the priming phase is released leading to unrestricted T cell activation [1].

**Figure 1:**
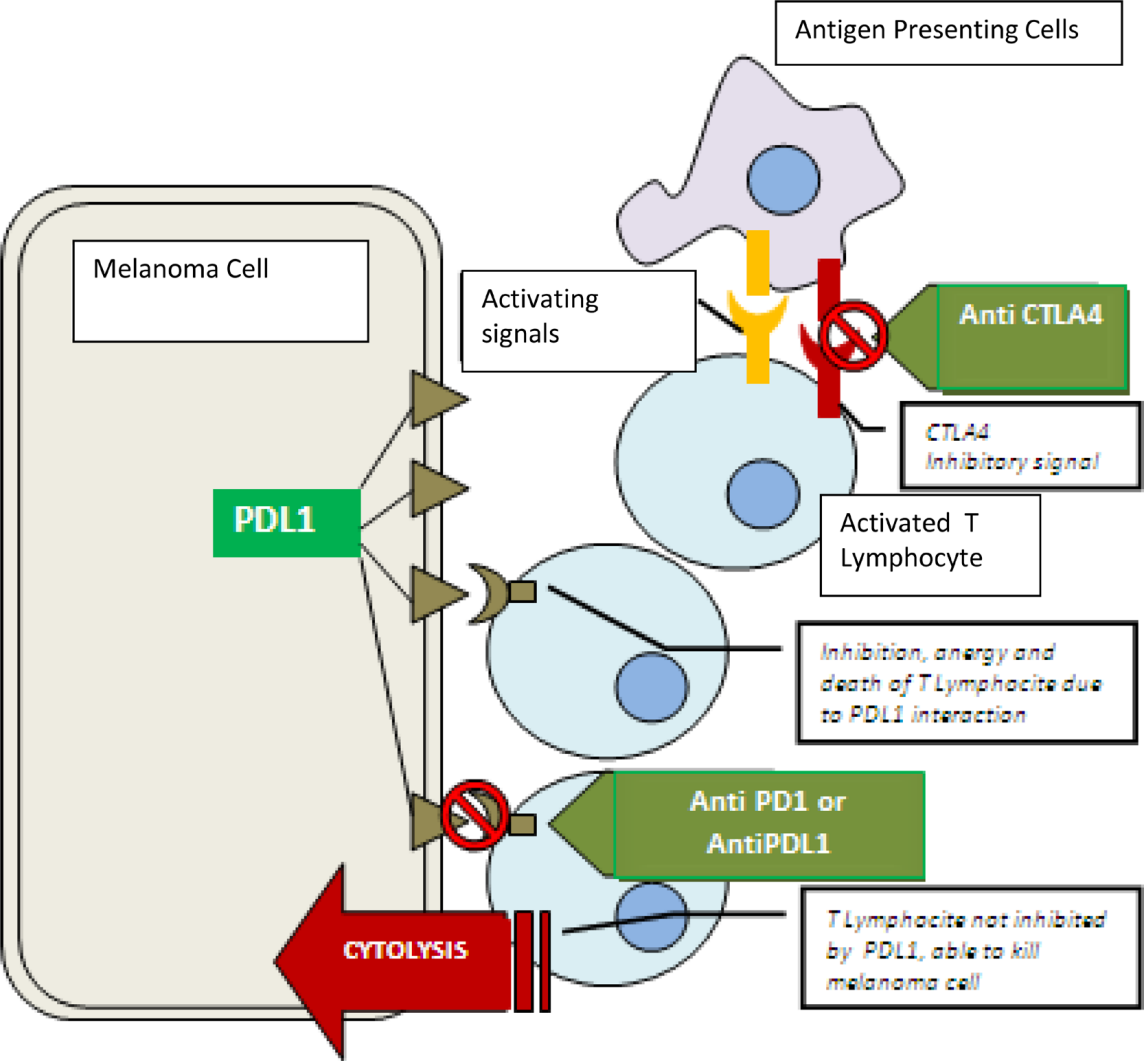
Mechanisms of action of anti-CTLA4, anti-PD-1, and anti-PD-L1 Antigen Presenting Cells interact with Lymphocyte: the immunological synapses is composed by activation signals (exemplified in yellow) and inhibitory signals like CTLA4 (exemplified in red).CTLA4 is a target of AtiCTLA4 drugs. Activated Lymphocyte migrate to melanoma cells. The interaction between PDL1 (expressed by melanoma cells) and PD1 (expressed by Lymphocyte) causes Lymphocyte anergy. AntPD1 and ANtPDL1 drugs prevent this interaction.

The PD-1 axis is another innate mechanism to reduce auto immunity and promote tolerance. Activated T cells express PD-1 receptor that, upon binding its ligands PD-L1 and PD-L2 (expressed in lymphoid cells, endothelial and epithelial cells, fibroblasts, dendritic cells, macrophages) induce T cell anergy. Melanoma cells express PD-L1 reducing the activity of infiltrating lymphocytes. By blocking the PD-1/PD-L1 interaction antitumor immunity can be restored [2–3] (Figure 1).

Prognosis of patients with metastatic melanoma dramatically improved due to the use of immunocheckpoints inhibitors. Still, some aspects need to be elucidated in the clinical application of these drugs, and a large field of research is involved in understanding how to raise the bar of efficacy of immunotherapy.

Starting with the current knowledge on immunomodulatory drugs currently available, this review aims to analyze controversial aspects such as optimal treatment sequences, research of biomarkers of efficacy, and innovative features, such as combinations of drugs currently under investigation.

### Biology of immune system

The immune system defends the organism from pathogens, and has a major role in fighting cancer development. It is divided in innate and adaptive immunity. Innate immunity is composed by white blood cells [granulocytes, macrophages, dendritic and natural killer (NK) cells] with phagocytic, cytotoxic and secretory activity; soluble factors including acute phase proteins and complement. Innate immunity acts in the first phases of the immune response, recognizing tissue damage and triggering the adaptive immunity. It also plays a role in the elimination of damaged cells. Adaptive immunity is mainly composed of Lymphosites. Antigen presenting cells (APC), such as dendritic cells, recognize stranger antigens from damaged tissue acquire the ability to migrate in lymphonodes where they present the antigens to T lymphocytes. The T cell receptor (TCR) recognizes protein-derived antigen that are assembled in the major histocompatibility complex (MHC-I or MHC-II) on the surface of the antigen-presenting cells (APC). Multiple costimulatory signals are needed to switch on the full activation of T lymphocytes: for example, CD28, a co-stimulatory receptor on the surface of T lymphocytes, binds to the B7 ligands, CD80 and CD86, on the surface of APC [110].

Activeted lymphocytes replicate and migrate to the dameged tissue. The CD4+ helper T cells and the CD8+ cytotoxic T cells (CTLs) are the main effectors against cancer cells: they manage humoral and cell-mediated response to kill cancer cells [8].

Some regulatory mechanisms are planned in order to control the intensity and duration of the T cell response or to mantein self tolerance: co-inhibitory immune checkpoint molecules, such as cytotoxic T-lymphocyte-associated protein 4 (CTLA4), can be overexpressed from lymphocytes to antagonize those costimulatory signals that activate lymphocytes; programmed death 1 (PD1) is another receptor expressed by lymphocytes that can lead them to exaustion when it recognizes its ligand; programmed death ligand (PDL)1 and PDL2 can be overexpressed by cancer cells. Other checkpoints include T cell Ig and mucin-domain-3-containing molecule 3 (TIM3), lymphocyte-activation gene 3 (LAG3) and killer cell immunoglobulinlike receptor (KIR) [10, 11]. Cancer cells use these molecules to evade the immune system. All in all, modern immunocheckpoints inhibitors aim to stop these inhibitory signals to restore lymphocyts activity against cancer [111].

### Immunocheckpoints agent: current status

Ipilimumab is the first and only anti-CTLA-4 drug approved by the FDA; it has demonstrated a survival at 3 years of 20% with similar results obtained regardless its use as first or second line treatment.

The first phase III trial was conducted in metastatic melanoma as second line treatment, and it compared ipilimumab 3 mg/kg every 3 weeks with GP-100 vaccine or the combination of both [4]. The study showed a significant improvement of survival (3.5 mo) for any of the ipilimumab-based arms. After a follow up of 2 years, overall survival was 13.7% for GP-100 monotherapy, 21.6% for the combined ipilimumab and GP-100 arms, whilst 23.5% for ipilimumab monotherapy, was also confirmed after a longer follow up period [5]. Soon after these promising results, ipilimumab was studied as first line treatment as an addition to standard DTIC chemotherapy at a dose of 10 mg/kg for 4 doses followed by maintenance every 12 weeks. Ipilimumab showed its superiority against DTIC: median overall survival was 11.2 months and 5 years survival rate was 18.2%, showing that efficacy was manteined in a subgroup of patients and observed also with longer follow up [6]. Tremelimumab is another anti-CTLA-4 monoclonal Antibody tested in metastatic melanoma patients. It is an IgG2 human mAb. In a phase III trial it was compared as a single agent to dacarbazine or temozolomide but it didn’t shows significant superiority in term of Overal Survival OS (11.8 vs 10.7 months respectively for the tremelimumab and for chemotherapy). It is still unclear why the results were so different, also because a longer follow-up at 2 and 3 years showed results very similar to those of ipilimumab (26.4% and 20.7%, respectively) [7].

The second class of inhibitors of immune checkpoints are the anti PD (Programmed Death)-1 agents. The PD-1 receptor, normally expressed by activated T cells, B cells, monocytes, and natural killer (NK) cells, is an inhibitory receptor activated by its ligands PD-L1 and PD-L2. PDL-1 is expressed in several cells, such as tumor cells and some host cells (myeloid, lymphoid, epithelial cells, antigen-presenting cells -APCs), so PD-1 works mainly in the tumor microenvironment [8, 9]. The interaction between PD-1 and PD-L1 inhibits the CD8^+^ cytotoxic T lymphocyte proliferation and survival, and induces TILs apoptosis and promotes differentiation of CD4^+^ T cells into Treg cells. Nivolumab and Pembrolizumab, the main anti PD-1 inhibitors studied, have been recently approved for the treatment of metastatic melanoma, with really promising results in terms of response rates and overall survival in different phase III trials.

Nivolumab is a fully human immunoglobulin G4 (IgG4) mAb anti-PD-1 agent. Several studies have been conducted both on BRAF v600 mutated and wild type patients. CheckMate 037 trial, 405 pretreated patients were enrolled, regardless of BRAF status, and randomized 2:1 to receive nivolumab (272 patients) or chemotherapy (133 patients) [10]: objective response rate (the primary endpoint) was 31.7% in the nivolumab group *vs*. 10.6% in the chemotherapy group. No statistically significant differences were observed in terms of median PFS in nivolumab vs chemotherapy arms (respectively 4.7 *vs*. 4.2 months). Grade 3-4 Adverse events incidence was lower in Nivolumab vs chemotherapy arm (respectively, 9% *vs*. 31%). Most frequent adverse events in the nivolumab group were pruritus, asthenia and diarrhea.

In the double-blind phase III trial CheckMate 066 418 untreated advanced BRAF wild-type melanoma patients were randomized to receive nivolumab or dacarbazine in first line. One year Survival rate was higher in nivolumab arm (72.9% *vs*. 42.1% - HR = 0.42; 99.79% CI, 0.25-0.73; *P* < 0.001) as median PFS was (5.1 *vs*. 2.2 months in the nivolumab armand in the dacarbazine arm respectively - HR = 0.43; 95% CI, 0.34–0.56; *P* < 0.001). The objective response rate was 40.0% in nivolumab group and 13.9% in the dacarbazine group (HR = 4.06; *P* < 0.001). Grade 3-4 adverse event incidence was only 11.7% [11].

Pembrolizumab is a humanized immunoglobulin G4 (IgG4) mAb anti-PD-1 agent. In the keynote-002 randomised phase II trial, 540 ipilimumab pretreate metastatic melanoma patients were enrolled: 180 patients were randomly assigned to receive pembrolizumab 2 mg/kg, 181 to receive pembrolizumab 10 mg/kg, and 179 to receive chemotherapy. Progression-free survival was improved in patients assigned to pembrolizumab 2 mg/kg (HR 0·57, 95% CI 0·45-0·73; *P* < 0·0001) and those assigned to pembrolizumab 10 mg/kg (0·50, 0·39-0·64; *P* < 0·0001) compared with those assigned to chemotherapy [120]. In the phase III clinical trial Keynote 006 trial was a Phase III clinical study in which 834 metastatic melanoma patients, were randomized 1:1:1 to receive Pembrolizumab 10 mg/kg every 2 weeks up to 2 years, *vs*. 10 mg/kg every 3 weeks up to 2 years, vs. ipilimumab 3 mg/kg every 3 weeks for 4 cycles. Patients were enrolled regardless of BRAF status, and 2/3 of them were treatment naïve. Median PFS was superior in pembrolizumab groups (5.5 months for pembrolizumab every 2 weeks, 4 months for pembrolizumab every 3 weeks and 2.8 months for ipilimumab, HR 0.58). Median OS was not reached in any of the treatment arms. One-year estimated OS rates were higher in the two pembrolizumab arms (74% for pembrolizumab every 2 weeks - HR = 0.63, 68% for pembrolizumab every 3 weeks - HR = 0.69, and 58.2% in ipilimumab group). At the pre-planned interim analysis the study was interrupted early for pembrolizumab superioriority in terms of Overal Survival, and cross-over was offered to patients on ipilimumab arm [13].

Several phase I studies evaluated activity of anti PD-L1 agents in melanoma cohorts. MPDL3280A, an IgG1engineered anti PD-L1 antibody reported a 39% ORR with a 43% 24-week progression free survival rate in 38 patients [15]. BMS-936559 a fully human IgG4 anti PD-L1 antibody achieved a 17% ORR in 52 patients [16]. Preliminary data about MEDI4736, an enngenireed IgG1 kappa monoclonal antibody, suggest that patients with melanoma remained in the phase I study beyond 12 week [17]. All these drugs showed modest toxicity profiles; attention is thus directed to their potential use in combinational regimens with other drugs commonly employed in melanoma treatment like BRAF and MEK inhibitors.

### Combination of anti-CTLA-4 and anti-PD-1

The rationale to combine anti-PD-1 and anti-CTLA-4 antibodies relays in their different mechanisms of action and their ability to modulate different phases of the interaction of tumor and immune system: anti-CTLA-4 acts mainly in the priming phase while anti-PD-1 blocks the effector phase in local tumor tissue.

The combination of nivolumab and ipilimumab has shown significant activity and has been currently approved by FDA for the first line treatment in advanced BRAF negative melanoma. In phase III CheckMate 067 trial, 945 previously untreated metastatic patients were randomized 1:1:1 to receive nivolumab monotherapy (dose 2 mg/kg every 14 days), nivolumab+ipilimumab combination (induction phase: nivolumab 1 mg/kg plus ipilimumab 3 mg/kg every 21 days for 4 cycles; mainainance phase: nivolumab 3 mg/kg every 14 days) or ipilimumab monotherapy (3 mg/kg every 21 days for 4 cycles); the study was designed to compare ipilimumab with the combination arm or with nivolumab. Co-primary endpoints were PFS and OS. Ipilimumab resulted inferior to both, achieving a median PFS of 2.9 months vs. 11.5 months (HR = 0.42; 99.5% CI, 0.31–0.57; *P* < 0.001) for nivolumab plus ipilimumab and 6.9 months (HR = 0.57; 99.5% CI, 0.43-0.76; *P* < 0.001) for nivolumab. The objective response rates were 43.7% in the nivolumab arm, 57.6% in the combination arm and 19% in the ipilimumab arm. The critical concern was toxicity: grade 3 or 4 AEs occurred in 55.0% in the nivolumab plus ipilimumab group *vs*. only 27.3% and 16.3% of patients in the ipilimumab group and in the nivolumab group, respectively. The most common AEs were diarrhea (8.3% *vs*. 4.5% *vs*. 1.9%, respectively) and colitis (8.3% *vs*. 7.7% *vs*.0.6% respectively) [12].

In order to reduce the incidence of severe adverse events, another combination regimens have been evaluated, with different drug dosage. In Keynote-029 pembrolizumab 2 mg/kg every 21 days was combined in the first 4 cycles with ipilimumab at a reduced dose of 1 mg/kg. Preliminary results showed high activity (PFS of 70% at 6 months), similar to that observed with nivolumab 1 mg/kg plus ipilimumab 3 mg/kg regimen, but with 25% immune-related grade 3-4 adverse events [14]. Other author evaluated this regimen in different treatment lines [121].

### Immune checkpoint inhibitors sequences: anti-CTLA-4 followed by anti-PD-1 and vice versa

Combined CTLA-4 and PD-1 inhibition is a valid therapeutic option for advanced melanoma. Phase 3 trials showed the superiority of such approach when compared to single agent immune checkpoint inhibitors [18]. Nonetheless, the serious adverse event (AE) rate is a definite issue (55% CTCAE G3-G4 AEs in the phase III trial). Theoretically, an alternative choice might be the sequential, rather than concomitant, administration of anti-CTLA-4 and anti-PD-1 monoclonal antibodies.

Anti-CTLA-4 agents can indeed upregulate PD-L1 expression, potentially enhancing the action of a subsequent PD1/PD-L1 inhibition in tumor microenvironment [19]. Nonetheless, patients with metastatic melanoma might need a rapid response, especially in presence of large tumor burden. In the adaptive immune response, CTLA-4 activation mediates an earlier phase than PD-1 one. In order to elicit an anti-tumor response, ipilimumab needs to activate T cells, while anti-PD-1 antibodies can activate lymphocytes directly in tumor microenvironment. Such a biological aspect has a direct clinical implication, because *in vivo* ipilimumab activity is slower than nivolumab or pembrolizumab one. Therefore, the upfront administration of anti-PD1 antibodies could lead to rapid responses, and sequential ipilimumab could result in enhanced therapeutic activity. Such approach could avoid the serious toxicities related to combined immunotherapy as well.

### Anti-PD1 followed by anti-CTLA4

Different retrospective trial ivestigated the role of ipilimumab after treatment failure to anti-PD1 therapy [122]. Aya et al. reported a case series of 9 patients treated with ipilimumab after progression on anti-PD1 antibodies. Two subjects (22%) had a partial response, while the remaining 78% (7 patients) experienced disease progression with a median a 3-month PFS and a 16-month OS. Serious AEs (≥ G3) were reported in five out of nine patients (55%) [20].

Another retrospective analysis was performed by Bowyer et al. on 40 melanoma patients treated with ipilimumab 3 mg/kg for 4 doses after progression to pembrolizumab or nivolumab. The objective response rate was 10%, but 35% of subjects experienced G3-G5 immune-related AEs. Therefore, ipilimumab is able to induce responses in patients previously treated with single agent anti-PD1 treatment, but the safety of such approach could be a concern [21].

### Anti-CTLA4 followed by anti-PD1

The reverse sequence, that is PD1 inhibition after progression on ipilimumab, was analyzed in retrospective studies. Shreders et al. described a series of 116 melanoma patients treated with pembrolizumab after anti-CTLA4 failure. Subjects experiencing disease progression at least 90 days after ipilimumab start had higher objective response and clinical benefit rates (ORR and CBR, respectively) when compared with patients progressing in the first 3 months of treatment (ORR 49% vs 35%; CBR 66% vs 46%). Moreover, outcomes with pembrolizumab were much better in subjects having a longer PFS (≥ 6 months) than in rapid progressors. Indeed, ORR and CBR were 55% and 80%, respectively, in long-term ipilimumab responders, whereas these rates were much inferior (25% and 25%, respectively) in rapid progressors (PFS < 45 days). [22]

Anti-PD1 after progression on ipilimumab was investigated in uveal melanoma as well. In a case series involving 25 subjects treated with pembrolizumab 2 mg/kg q21days, median PFS was 91 days and median OS was not reached after a median follow-up of 32 weeks. Serious (G3-G4) AEs were observed in 25% of patients (5/25) [23].

The only prospective trial studying immune checkpoint inhibitors sequences was published in 2016. Weber et al. conducted a randomised, open-label, phase 2 study aimed at evaluating the sequencing treatments with ipilimumab and nivolumab. 140 patients were randomly assigned to induction with nivolumab 3 mg/kg every 14 days for 6 doses followed by a planned switch to intravenous ipilimumab 3 mg/kg every 21 days for 4 doses, or the reverse sequence; after this first phase, both groups received intravenous nivolumab 3 mg/kg every 2 weeks until progression or unacceptable toxicity. During the whole study period, nivolumab followed by ipilimumab lead to a higher incidence of adverse events (63% G3-G4 AEs) than the reverse sequence (50% G3-G4 AEs). Nevertheless, the former sequence was associated with a higher response rate than the latter (35% vs 10% at week 13; 41% vs 20% up to week 25) [24].

Both FDA and EMA approved ipilimumab, pembrolizumab and nivolumab as single agents, as well as ipilimumab and nivolumab in combination. Further prospective randomized studies are to be performed in order to evaluate the effectiveness and the safety of sequential anti-CTLA4 followed by anti-PD1 or vice versa. In fact, the optimal sequential approach remains an unmet clinical need, especially for patients unfit for the combination.

### Tissue biomarkers

In order to detect patients that benefit from immune checkpoint inhibitors, several trials investigated potential tissue and circulating biomarkers. PD-1 and PD-L1 expression, tumour-infiltrating lymphocytes, the T-cell receptor repertoire, and mutational or neoantigen burden are the most studied biological tissue characteristics, but characterization of the tumor microenvironment immune state still needs to be improved. Further investigation into the relationships between all these aspects should be aimed at creating an optimized model for predicting response to anti-PD-1 or anti-PD-L1-based therapies.

PD-L1 expression on melanoma cells has been the first candidate as a biomarker for anti-PD-1 drugs, but in melanoma it has no established role: many studies evidenced a high proportion of objective response in patients that resulted PDL1 negative; also PFS and OS resulted improved irrespective of PDL1 expression. In Keynote -001 trial patients affected by metastatic melanoma with different grade of PD-L1 expression and treated with pembrolizumab showed a variable reduction in tumor dimention, with renge from 35% to 86%: even if it seems that lower PD-L1 expression correlate with lower response, the high rate of response doesn’t allow to prevent this group of patients from immunocheckpoints treatment. [123] The poor reliability of PD-L1 immunohistochemistry as a biomarker for anti-PD-1 or anti-PD-L1 therapies is probably the result of multiple variables: PD-L1 expression is actually regulated by various mechanisms, including the MAPK and PI3K or Akt pathways, transcriptional factors HIF1, STAT3, and NFkB, and epigenetic factors [25]. It can be also expressed by immune cells in the tumor microenvironment. PD-L1 expression can be transient, and intrapatient and even intratumour heterogeneity in PD-L1 tumor expression can exist [26] Eventually, different detecting antibodies, thresholds for positivity, and quantification techniques have been used, so data are hardly comparable among trials [27].

Several studies identify a role of tumor infiltrating lymphocite as a marker of activity of immunomodulating agent: its predictive role is discussed, because baseline CD8+ T-cell density overlapped between the patients with a response and those with disease progression. However, it is interesting to note that modification of infiltrating lymphocite density from margin to tumor improved in an higher percentage in responsive disease, both in ipilimumab [28] and in antiPD1 trial [29].

In a small study conducted on 23 patients treated with anti PD-1 pembrolizumab, next-generation sequencing was done on pretreatment melanoma tumors to capture all uniquely rearranged variable T-cell receptor β-chain regions. The study showed that the presence of a more clonal T-cell population correlates with benefit from anti-PD1 treatment more then heterogeneous T cell population. In fact, T-cell receptor β chain usage was more restricted in the responding patient group than in those with disease progression [28].

Schumaker et al classified all tumor types based on mutational load and postulated that tumor types with high mutational burdens are more responsive to immunotherapy strategies. Melanoma is located at the top of the classification: it has a high median mutational load, the greatest number of neoantigens, and it is responsive to checkpoint immunotherapies [30].

Multiple studies in different cancers corroborate the relationship between tumor load and immunosensibility. In melanoma, a study by Snyder and colleagues [31] showed a better clinical benefit from anti CTLA-4 therapies (ipilimumab or tremelimumab administered in 64 patients) in melanoma patients with a mutational load of more than 100 non-synonymous somatic mutations based on tumor whole-exome sequencing. This mutational load cutoff was associated with longer overall survival compared with patients with a lower mutational load (*p* = 0.04 in the discovery set and *p* = 0.01 in the validation set by log rank test). Furthermore the study underlines the importance of neoepitopes: a neoepitope signature based on major histocompatibility complex (MHC) class I presentation was highly associated with clinical outcome with overlap in neoepitopes predicted to occur in many responding patients. It seems that the mutational load gives more immunosensibility in proportion of a higher probability to induce immunogenic neoepitope through passenger mutations. Also anti PD1/PDL1 drugs have been correlated with mutational load [32]. 65 patients with advanced melanoma were treated with nivolumab or pembrolizumab or atezolizumab and High mutational load, measured by hybrid capture-based next-generation sequencing, was associated with response to therapy and long median progression-free survival and overall survival.

Expression of immune-related genes can discriminate groups of melanoma responsive to immunotherapy. Studies of gene expression profiling showed that melanoma treated with immunocheckpoint inhibitors showed better treatment efficacy, if they expressed genes related to inflammatory response. In a retrospective analysis [33] of patients with advanced melanoma given ipilimumab in a phase 2 clinical trial (CA184004) the expression of 22 immune-related genes had at least a 2.5-times increase in responders. This gene profile included markers for cytotoxic T cells (eg, CD8A, granzyme B, perforin 1), Th1 cytokines or chemokines, MHC class II (HLA-DQA1), and other immune-related genes (eg, *NKG7*, *IDO1*). An interferon γ score was developed by Ribas and collegue [34]. These scores showed significant correlation with best overall response and progression-free survival to anti PD1. The gene score included those encoding interferon γ (*IFNG*), granzyme A and B (GZMA and GZMB), and perforin 1 (*PFR1*), *IDO1*, *LAG3*, and other immune-related genes. In support of the importance of IFNy gene signature, Johnson and colleagues [35] showed that high MHC class II (HLA-DR) expression was associated with improved clinical response, longer progression-free survival, and longer overall survival in patients with melanoma given anti-PD-1 or anti-PD-L1 therapy.

### Circulating biomarkers

Blood-derived parameters have been correlated with survival of melanoma patients treated with antiCTLA4, including baseline absolute neutrophil count (ANC) or the neutrophil to lymphocyte ratio (NLR), known markers of systemic inflammation. The data from the Italian expanded access program with ipilimumab at 10 mg/kg demonstrated an increase in the number of circulating ICOS+T cells at week 7 in parallel to disease control and improved survival [36]. Increase of ANC observed 2 to 8 weeks after initiation of Ipilimumab and expansion of activated T cells reflect change associated with positive outcome [37].

An increase in the eosinophil count > 100/mm^3^ between the first and the second infusions and a lymphocyte count >1000/mm^3^ at the start of the second course were associated with an improved OS [38]. High NLR (neutrophil/lymphocyte) ratio, high ANC (> 2), before initiating ipilimumab treatment in patients with metastatic melanoma are independent prognostic indicators of poor survival [39].

Moreover, the derived NLR (dNLR), composed of white cell counts (WBC) and ANC, has been proposed as an alternative to NLR to detect a potential biomarker of response to ipilimumab. Prospectively collected data from 720 advanced melanoma patients treated with ipilimumab 3 mg/kg within Italian EAS were analyzed. Baseline ANC and derived neutophil to lymphocyte ratio (dNLR) were associated with outcome of ipilimumab melanoma patients. Patients with both ANC > 7500 and dNLR > 3 had a significant increased risk of death and a progression compared with patients with lower ANC and dNLR. The 1 and 2 year survival rate were 2% and 0% respectively, for patients with ANC > 7500 and dNLR >3 and 43% and 24%, respectively, for patients with both lower ANC and dNLR [40].

Since LDH levels and neurophil count were independent prognostic factors in melanoma, a study showed that ipilimumab may be the best treatment in patients with high neutrophil count and LDH, in particular when superior to 7.5 × 106/l and x 2 upper normal limit (UNL), respectively [41].

It was postulated also the evaluation of melanoma markers on circulating cells as Melan A, gp100, MAGE3, MIA (melanoma inhibitory antigen) prior to the treatment and within the therapy were compared to the data collected at baseline after the melanoma surgery. Lower levels were linked to longer survival time. A reduction by 30% at week 6 to week 9 of ipilimumab administration was associated with response to therapy [42].

A significant decrease in myeloid-derived suppressor cells (MDSC) as immune-regulatory cells in concomitant with increase of fully activated type-1 CD4+/CD8+ T cells and improved progression free survival was described (PFS) [43].

During ipilimumab treatment, MDSC frequencies did not change compared to baseline levels. MDSC frequencies in ipilumumab-treated patients were independently of baseline serum lactate dehydorgenase levels but tended to increase in patients with metastasis in skin or lymph nodes only (M1a). Clinical responder to ipilimumab therapy showed less of MDSC as compared to non-responders. These data suggest that the frequency of MDSC may be used as a predictive marker of response while low frequencies identify patients more likely benefitting from ipilimumab treatment. Briefly, during treatment with Ipilimumab, disease control and survival were significantly associated with decreasing levels of lactate dehydorgenase [44], C-reactive protein, FOXP3 regulatory T cells (Tregs), lower pretreatment level of circulating MDSC increasing absolute lymphocyte count between baseline and end of treatment [45], increase of ANC and CD4+/ICOS^high^ T cells [46].

Studies to identify peripheral blood immune biomarkers during anti-PD1 treatment illustrated that PD-1/PD L1 blockade increases effector T-cell proliferation (CD8+/HLA-DR+/Ki67+ T cells), production of interferon-gamma (IFN-γ) and IL-18, but without significant correlation with clinical response in patients. A better clinical response to pembrolizumab was noticed in melanoma patients who had TCR oligoclonality and a higher number of baseline CD8+ T cells. Moreover, tumor mutational load could be potential predictive biomarker for PD-1/PD- L1 blockade therapy: it is demonstrated that tumors with high mutational load are likely more immunogenic, due to higher production of neoantigens and consequent stimulation of neoantigen-specific CD4+ and CD8+ T cells [47].

Recently, a framework is proposed for describing the different interactions between cancer and the immune system in individual cases, with the aim to focus biomarker research and to help guide treatment choice. The outcome of cancer-immune interactions is based on a number of largely unrelated parameters such as tumor “foreignness” and T cell–inhibitory mechanisms. Seven parameter classes may constitute a reasonable initial framework for building the so-named “Cancer immunogram”, including the evaluation of tumor mutation load, lymphocyte count, intratumoral T cells, PD-L1 expression on tumor, serum levels of c-reactive protein (CRP), LDH and IL-6. Thus, the information obtained from the combination of tumor genomics, immunohistochemistry, and standard assays on the peripheral blood compartment to visualize the state of cancer-immune interactions in individual patients could help clinicians to define treatment options in a more refined and personalized manner [48].

### New combinations on the horizon

**Figure 2:**
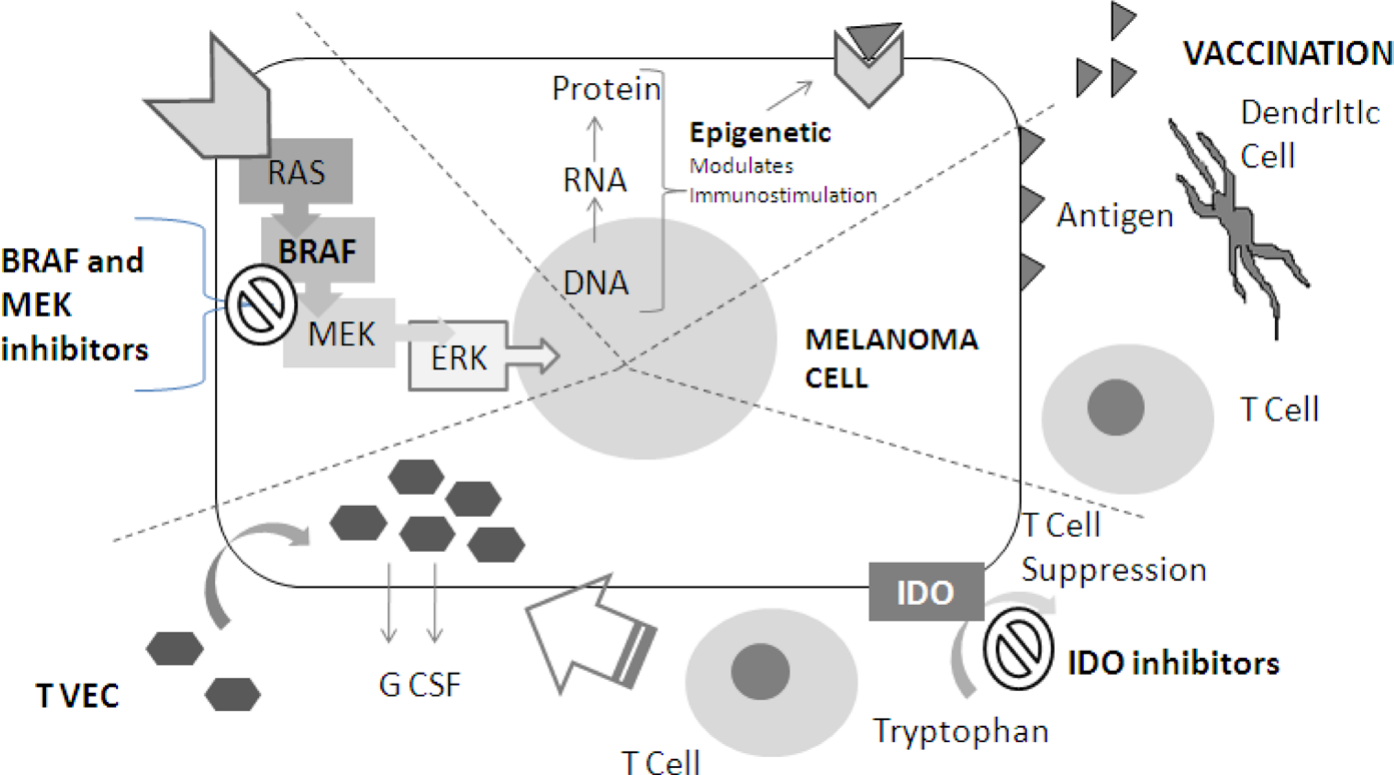
New Agent for combinations with immunotherapy: meccanism of action (1) BRAF end MEK inhibitors block MAPKinasi pathway (indicated with RAS, BRAF, MEK, ERK in the figure). (2) Epigenetic drugs modulates proteins expression trough an alteration in usual DNA-RNA-protein sequence, and causing immnostimulation (see text). (3) Antigen (exemplified with triangles) vaccination: Vaccination stimulate dendritic cells to activate immunoresponse against melanoma cell. (4) IDO inhibitors repress T cell suppression by blocking tryptophan conversion. T VEC therapy cause local immunoinfiltration by causing local G CSF production.

In order to improve the activity of immune-checkpoints inhibitors, their combination with different agents seems a promising strategy. Agents with different mechanisms of action and different safety profiles may potentially have a therapeutic synergistic effect in several cancer types or even overcome mechanisms of resistance [85–86]. Several trials studied the combination of immunocheckpoints inhibitors and other systemic treatment, such as vaccines, epidrugs, targeted therapies, different immunotherapies, (see Figure 2) and chemotherapy.

### Immune check points inhibitors and vaccines

The rationale of developing anticancer vaccines dates back to the 1950s, when the presence of cancer-specific antigens potentially inducing an immune response was first observed [49]. Since then many clinical trials of therapeutic vaccination have focused on melanoma, but results have been disappointing: indeed, the MMAIT-IV phase III study unexpectedly showed worse survival in the vaccine arm with respect to placebo [50], while other randomized trials have not showed any benefit for various immunologically effective vaccines [51], despite they were all immunologically effective, i.e. capable of inducing an antigen-specific T-cell response.

Overall, these data suggest that the antitumor immunity developed after anticancer vaccination with current techniques is largely ineffective and not sufficient to obtain a clinically meaningful benefit in the majority of patients. Immune checkpoint inhibitors counteract some physiologic mechanisms suppressing the immune response (among others CTLA-4, PD-1/PD-L1, LAG-3) and therefore, could be employed to boost a highly specific, vaccine-induced anticancer response, potentially increasing efficacy. This strategy is also supported by preclinical data demonstrating enhanced vaccination-induced priming of T cells after CTLA-4 blockade [52]. The first large-scale clinical trial evaluating this strategy was the MDX010-20 study, which compared the combination of ipilimumab plus gp100 vaccine with ipilimumab alone or gp100 alone. Both ipilimumab arms resulted superior to gp100 alone, without any meaningful difference between them [53].

Another smaller, phase II study in adjuvant setting evaluated the combination of ipilimumab with a multipeptide vaccine (tyrosinase, gp100 and MART-1), in view of the multiplicity of antigens potentially expressed by melanoma. Anyway the vaccine failed to show any significant clinical activity, and immunological activity was also disappointing: only 25% of patients developed immune response against gp100 or MART-1, and none to tyrosinase [54].

An alternative approach focused on the immunosuppressive role of indoleamine 2,3-dioxygenase (IDO), identified as one of the mechanisms of resistance to ipilimumab. In an attempt to target the enzyme, a small phase I clinical trial employed a combination of a peptide vaccine from IDO and ipilimumab. An immune response to the vaccine was detected in 3/10 patients and 5/10 obtained a stable disease at first evaluation, suggesting that efficacy might not be superior to ipilimumab alone. The authors also reported a death presumably related to treatment-induced colitis.

PD-1 blocking antibodies have also been evaluated in combination with vaccines. Preclinical evidence actually showed vaccine-dependent PD-L1 upregulation in the tumor microenvironment, thus supporting this strategy [55]. A first trial evaluated a combination of nivolumab and a multipeptide vaccines targeting gp100, MART-1 e NY-ESO-1 in 90 patients with pretreated melanoma, either ipilimumab-naïve or refractory. Overall response rate was 25%, with no difference related to previous ipilimumab administration, and disease control rate was 46%. Responders had less CD8+ cells specific for NY-ESO-1 and MART-1 in the peripheral blood at baseline, whose increase after vaccination was not statistically significant [56]. A second trial evaluated the same combination as adjuvant therapy after complete resection for stage IIIC or IV melanoma. After treatment, patients showed increased peripheral CD8+ T cells specific for the vaccination antigens, suggesting that the vaccine may be more immunologically active in the adjuvant setting. From the clinical standpoint, the relapse rate of 30% after a median follow-up of 32.1 months may be encouraging, but further studies are needed to discern the relative contribution of vaccine and nivolumab, if any, to this result [57].

In summary, the studies conducted so far have shown that adding a peptide vaccine to the currently available immunomodulating antibodies is generally safe, but does not result in increased clinical activity or efficacy. However, peptide-based vaccines may be suboptimal: some studies actually suggest that dendritic cell (DC) vaccines may be more effective both immunologically and clinically [58]. To be used as a vaccine, DCs are generally obtained *ex vivo*, loaded with desired tumor antigens by different techniques, and finally administered to the patient. Such products, as conventional vaccines, are remarkably safe and immunologically effective [59]. Anyway, despite long-lasting clinical responses and encouraging survival have been reported [60], a single, small randomized trial failed to demonstrate superiority of a dendritic cell vaccine over dacarbazine in metastatic melanoma [61]. The sole clinical trial evaluating a combination of a DC vaccine with ipilimumab in 39 pretreated advanced melanoma patients, however, showed promising results: 51% of the patients were progression-free at 6 months, with an objective response rate of 38%. Immune-related toxicity was not marginal, but manageable and reversible with steroid therapy [62]. These preliminary results suggest that the combinations of DC vaccines and immune checkpoint inhibitors deserve further evaluation in prospective clinical trials.

### Immunocheck points inhibitors and epidrugs

Epigenetic is the field of biology that studies all the heritable and potentially reversible changes in gene expression that occur without altering DNA sequence [63]. The three main epigenetic fields are the following: microRNA, DNA methylation, histone modifications.

MicroRNAs (miRNAs) are small single-stranded RNA molecules; they usually are of 22 nucleotides in length. MiRnas regulate gene expression modulating the post-trascription process. [64]

DNA methylation occurs in the so called “CpG islands” that are part of the promoter region of genes and that are compoused by CpG dinucleotides. Methylation is catalyzed by DNA methyltransferase (DNMT) enzymes. Methylation levels induce different accessibility to gene transcription.

Histones are the primary component of chromatin; their function is organization of DNA into nucleosomes. Accessibility of genes to transcription factors is regulated by post-translational covalent histone modifications [65] which include methylation, phosphorylation and acetylation (that is catalyzed by histone acetyltransferases and deacetylases - HDACs). This last field is the most exploited in melanoma.

All epigenetic changes induce also an immunomodulation of the interaction between tumor and immune system. An increasing number of data shows that epigenetic drugs have a role in facilitating immunological targeting of cancer cells by modulating different molecules and pathways that mediate the interaction between the immune system and cancer cells.

HDAC and DNMT inhibitors can have some immunomodulatory effect: for example, they can result in altered antigen presentation of PD1 and MHC and elevated immunogenicity as evidenced by increased expression of CD25, CD40 or CD80 and other costimulatory molecules; they can alter the FoxP3 expression, the function of DC, increase the activity of CDb+ lymphosites and decrease the activity of CD4*lymphosites. Further, they can induce G1 cell cycle arrest with an increase in cyclin D1 impaired cell proliferation groth reduction and induction of apoptosis.

Thus, epigenetic drugs seem an option to overcome some limitations of currently available immunotherapeutic regimens and a strong rationale exists to use them in combination. A second generation epigenetic drugs with improved efficacy and clinical tolerability are now being developed. Preclinical data support the feasibility and activity of the proposed strategy. In particular, systemic administration of 5-AZA-CdR proved effective in modifying the immune phenotype of human metastatic melanoma xenografts, by inducing or up-regulating cellular CTA expression and expression of HLA class I antigens and HLA A1 and A2 allospecificities [66]. These *in vivo* modifications were remarkably durable; NY-ESO-1 expression and HLA class I antigen up-regulation were still detectable on melanoma xenografts 30 days after the end of 5-AZA-CdR administration. Emphasizing the notion that epigenetic modification of tumor cells strongly up-regulates their immunogenicity, injection of BALB/c mice with 5-AZA-CdR-treated human melanoma cells generated high-titer anti- NY-ESO-1 antibodies [66]

### Combinations of immune-checkpoints inhibitors and targeted therapies

Pre-clinical studies showing that combination of BRAF and MEK inhibitors has immuno-modulatory properties and may enhance immune activation [67–71] led to the investigation in clinical trials of regimens including MAPK inhibitors targeted therapies and immune-checkpoint inhibitors (Table 1).

**Table 1:** Selected melanoma clinical trials exploring the combinations of immune-checkpoint inhibitors with BRAF/MEK targeted therapy (see attatched file)

Study	Phase	Status	Treatment	Strategy
NCT01400451	I	Terminated due to dose limiting toxicities	Ipilimumab Vemurafenib	Anti-CTLA-4 + BRAFi
NCT01767454	I	Completed	Ipilimumab Dabrafenib +/- Trametinib	Anti-CTLA-4 + BRAFi+MEKi
NCT02027961	I	Active, not recruiting	MEDI4736 Dabrafenib +/- Trametinib	Anti-PD-L1 + BRAFi+MEKi
NCT01656642	Ib	Recruiting	Atezolizumab Vemurafenib +/- Cobimetinib	Anti-PD-L1 + BRAFi+MEKi
NCT02130466	II	Recruiting	Pembrolizumab Dabrafenib + Trametinib	Anti-PD-1 + BRAFi+MEKi
NCT02908672	III	Not yet recruiting	Atezolizumab Vemurafenib +/- Cobimetinib	Anti-PD-L1 + BRAFi+MEKi

Early phase clinical trials combining ipilimumab and BRAF/MEK inhibitors revealed severe toxicities. The phase I trial of ipilimumab plus vemurafenib (NCT01400451) was terminated early due to hepatotoxicity [72] and the phase I trial of ipilimumab plus dabrafenib and trametinib was suspended after 2/7 patients enrolled developed colitis with colonic perforation [73]; in the same study, no dose-limiting toxicities were observed in the cohort treated with ipilimumab plus dabrafenib.

PD-1/PD-L1 checkpoint inhibitors are associated with less severe toxicities than anti-CTLA-4 [74] thus making them ideal candidates to explore combinations with BRAF/MEK targeted therapies. In a phase I study of the PD-L1 inhibitor MEDI4736 given in triple combination with dabrafenib and trametinib (NCT02027961), only 3/26 patients receiving such regimen discontinued study treatments due to an adverse event, with no toxicities leading to death; dose-limiting toxicities were observed in only one patients treated with dabrafenib+trametinib+MEDI4736 (reversible grade 3 thrombocytopenia). Despite the limitation due to the small number of patients, such triple combination showed promising clinical activity, with 69% of patients achieving a response and 100% achieving disease control [75].

Similar results were obtained in a phase Ib dose-escalation study of vemurafenib plus atezolizumab (NCT01656642). Notably, in such study, a lower proportion of patients treated with a 28-days run-in treatment with only vemurafenib followed by concurrent administration with atezolizumab had grade 3 toxicities compared with patients who received a front-line concurrent vemurafenib+atezolizumab treatment [76]. An additional cohort with a 28-days run-in treatment with vemurafenib and cobimetinib followed by concurrent triple administration with atezolizumab led to the opening of a large phase 3 randomized, placebo-controlled study (NCT02908672) to assess the efficacy of the triple combination compared with vemurafenib and cobimetinib only.

Pembrolizumab plus dabrafenib and trametinib is another triple combination assessed as a tolerable treatment in a phase I, dose-identification study, despite a considerable rate of grade 3-4 adverse events (67%) and discontinuation due to toxicity (33%) [77]. With a promising clinical activity being observed in the phase I study, such regimen is currently under investigation in a randomized, placebo-controlled phase II clinical trial (NCT02130466).

Immune checkpoint inhibitors and BRAF/MEK targeted therapy combinations showed promising clinical activity in early phase clinical trials, but at an increased rate of severe toxicities; therefore, such treatments may not be the best approach or suitable to all patients, and efforts should be addressed to the identification of predictive factors selecting patients at increased risk of severe toxicities.

As long as the combined treatment of immunocheckpoints inhibitors and targeted therapies will remain an experimental option, in clinical practice the controversy remains over which is the best first-line choice in the BRAF mutated metastatic patient between immunotherapy and target therapy. If historically targeted therapies offered rapid shrinkage without long-term survival, today with the combination of MEK and BRAF inhibitors, Overal Survival curves are observed with a large share of long survivors [113], and it is evident that patients who benefit best from this strategy are low-tumor burden and low basal LDH patients [112]. If initially antiCTLA4 provided long-lasting survival for a minority share of patients at the expense of rapid progression of many, to date with the anti-pd1 and the combination of antiCTLA4 and anti PD1, the proportion of long surviving patients has increased considerably as the rapidity of achieving the answer [12]. The discussion is open and clinical and translational research will be the key to such questions.

### Combinations of immune-checkpoints inhibitors and other immunotherapies

The combination of ipilimumab and nivolumab achieved higher clinical activity at the cost of significant toxicities. In addition to studies with anti-PD-1 in combination with low dose ipilimumab, other combination regimens are currently under investigation to achieve better efficacy outcomes with a lower impact on toxicity.

The combination of pembrolizumab and epacadostat, an orally available inhibitor of indoleamine 2,3-dioxygenase (IDO1), was well-tolerated and achieved impressive results in a phase I study. IDO1 is a tryptophan-catabolizing enzyme that is overexpressed in many cancers and induces immune tolerance by suppressing T-cell responses; IDO1 inhibition exhibits antitumor activity through the reactivation of effector T cells and is synergistic with PD-1 blockade [78–83] Such combination is currently being investigated in a randomized, placebo-controlled phase III clinical trial (NCT02752074) [78].

Talimogene laherparepvec (T-VEC), a herpes simplex virus type 1–derived oncolytic immunotherapy designed to selectively replicate within tumors and produce granulocyte macrophage colony-stimulating factor (GM-CSF) to enhance systemic antitumor immune responses, produced durable responses and a therapeutic benefit against melanoma in a phase III clinical trial [84], and was recently approved by the FDA for the treatment of melanoma lesions in the skin and lymph nodes. Despite a lower clinical activity and efficacy than anti-PD-1 drugs, T-VEC demonstrated to be a very tolerable treatment, with only 4% patients discontinuing therapy as a result of adverse events in the phase 3 trial; therefore, it may represent a valuable option for combination therapy. The safety and activity of the combination of pembrolizumab and T-VEC, which increases tumor-derived antigen expression and T cell infiltrate and may act in synergy with PD-1 blockade, was explored in a phase Ib study: the combination was well tolerated, with no DLTs being observed and no patients discontinuing treatment due to treatment-related adverse events, and active, with an overall response rate of 56% [85–86]. Such results supported the conduction of a randomized, placebo-controlled phase III trial to assess the efficacy of the combination of pembrolizumab and T-VEC compared with pembrolizumab as single agent (NCT02263508).

### Immunocheckpoints inhibitors and chemotherapies

The strategy of combining chemotherapy and immunotherapy is based on the notion that combination regimens may potentiate the immune system to more easily recognize neoantigens released upon cytotoxic chemotherapy-induced tumor cell death [87–88].

The combination of chemotherapy and cytokines immunotherapy was variously assessed in several melanoma clinical trials, but failed to provide improved survival, despite a higher rate of responses compared to chemotherapy alone [89].

In spite of the lack of evidence showing an improvement of survival in metastatic melanoma patients, dacarbazine has been the drug most frequently used and compared, both as single agent or combined with other therapies in randomized studies [90–91]. Recently, immune-checkpoint inhibitors have become widely available for the treatment of advanced melanoma and the combination of such drugs with chemotherapy have been investigated. First, the combination of ipilimumab with dacarbazine was evaluated in a phase II study of ipilimumab 3 mg/kg plus dacarbazine 250 mg/m2 versus ipilimumab alone (MDX010-08). It was observed that adding dacarbazine to ipilimumab did not suppress but instead enhanced the effect of ipilimumab [92].

Five-years follow up of the phase III trial CA184-024, which compared ipilimumab at 10mg/kg plus dacarbazine 850 mg/m^2^ with placebo plus dacarbazine in treatment-naïve patients with advanced melanoma showed an improvement in median overall survival for the combination arm with 11.2 versus 9.1 months (hazard ratio [HR], 0.72; 95% CI, 0.59 to 0.87; *P* = 0.001) [93]. No new toxicities were reported; however, severe liver toxicity was observed in the combination arm, with high rates of G3-4 adverse events, probably due to the additional hepatotoxicity of dacarbazine [94–96]. The 5-year survival analysis confirmed that approximately 20% of treated patients achieve long-term survival, similarly to rates observed for ipilimumab as single agent.

The combination of ipilimumab and fotemustine was also evaluated. Fotemustine, a nitrosurea alkylating agent able to pass the blood-brain barrier, has shown activity in patients with brain metastasis. In a phase III trial, a trend in favor of fotemustine was observed in terms of overall survival and, notably, a better time to BM was evidenced compared to dacarbazine in patients with stage IV melanoma [97]. An Italian phase II, open label, single arm study (NIBIT-M1) aimed at assessing the efficacy and safety of the combination of ipilimumab 10 mg/kg plus fotemustine 100 mg/m^2^ in patients with stage IV melanoma, including patients with asymptomatic brain metastasis. The combination of ipilimumab plus fotemustine had clinical activity in a subset of patients with or without brain metastasis, despite greater toxicities (more than 50% of treated patients experienced G3-4 drug related adverse events) [98]. Ipilimumab plus fotemustine showed a clinical benefit in patients with brain metastasis. The combination seemed also to prevent or postpone the appearance of brain metastases.

A three-year survival analysis showed similar outcomes in patients with or without brain metastases, further supporting that such a combination might be effective in patients with NCS involvement. Median OS was 12.9 months [95% confidence interval (CI), 7.1–18.7 months] for the whole study population, and 12.7 months (95% CI, 2.7–22.7 months) for patients with brain metastases, respectively. The three-year survival rates were 28.5% for the whole study population and 27.8% for patient with brain metastasis [99].

After the encouraging results obtained with the combination of ipilimumab plus fotemustine, in particular in patients with brain metastasis, a second randomized phase III study (NIBIT-M2) comparing fotemustine 100 mg/m^2^ vs fotemustine 100 mg/m^2^ plus ipilimumab 10 mg/kg or ipilimumab 3mg/kg plus nivolumab 1 mg/kg) was initiated in order to evaluate the safety and specifically OS in patients with brain metastasis.

So far the combination of immune-checkpoint inhibitors and chemotherapy has not yielded the expected results. However, the strong rationale supporting this strategy and the partial successes in specific subgroups of patients encourages further investigation.

### Abscopal effect and immunosystem

The abscopal effect of radiation is a rare phenomenon that occurs when tumor regression is observed in non-irradiated tumor sites after a local application of radiotherapy [100–101]. Studies reported that radiation improves antitumor response to immunotherapy through several mechanisms: enhancement of the major histocompatibility complex class I, expression of calreticulin and factor for surface apoptosis signals; activation of dendritic cells; enhanced cross-presentation of tumor antigens; increased density of tumor-infiltrating lymphocytes; changes in expression of immune checkpoint molecules; and modulation of Treg cell populations (101). Radiotherapy-induced cell death is a potential source of tumor antigens, which may indeed be uptaken, processed, and presented by dendritic cells to CD8+ T lymphocytes.

Another possible explanation of the synergy between radiotherapy and immunostimulatory mAbs is that radiotherapy seems to induce a more intense expression of CD137 and/or PD1 on tumor-infiltrating T lymphocytes; moreover, radiotherapy causes vascular inflammation and activation of antigen-presenting dendritic cells (DCs) [102].

Systemic, immune-mediated abscopal effects on tumor regression have been detected in preclinical and early clinical trials because of the intensifying T-cell effects of the combinatorial therapies [100]. The combination of irradiation with immunotherapy may increase the occurrence of abscopal effect, with rates of 25%–52% with immune checkpoint inhibitors [101].

### Abscopal effect and anti-CTLA-4

Anecdotal evidence suggested that in patients treated with anti CTLA-4 mAb (ipilimumab) and subsequent palliative radiotherapy objective responses were detected outside the irradiation field, concurrent with increases in the titer of antibodies against the shared tumor antigen NY-ESO1 [103].

It has been speculated that such an antigen-presenting cell subset is the main mediator of productive tumor antigen presentation to CD8+ T cells and the IFNa/b levels are critically involved in the radiotherapy-induced abscopal effects [104]. Genomic DNA released by dying tumor cells is probably involved in eliciting IFNa/b via endoplasmic-reticulum-associated protein STING (stimulator of IFN genes) and, in turn, IFNa/b may act on both cross-priming DCs and CD8+ T cells to favor, as a necessary factor, the immune response by T cell line. Strategies aiming at local enhancement of IFNa/b could render radiotherapy-induced tumor cell death more immunogenic as recently shown for chemotherapy [104].

The first case reported of abscopal effect was a patient with metastatic melanoma who underwent maintenance with ipilimumab and concurrent palliative radiotherapy (28.5 Gy) at disease progression resulted in regression of non-irradiated metastases that showed benefits for at least 10 months [105].

Hiniker and colleagues discussed to combine ipilimumab and concurrent radiotherapy for a patient with asymptomatic melanoma [106]. That patient was given a higher radiation dose (54 Gy in three fractions) and showed a complete response in both the primary tumor and the metastatic lesions, which confirms the findings from pre-clinical studies indicating the importance of radiation dose [107].

Many other reports also observed benefit of radiotherapy combined to ipilimumab in metastatic melanoma or NSCLC, including patients with complete responses [102].

Of note, a series enrolling 21 patients with advanced melanoma who progressed after ipilimumab and then underwent radiotherapy for cranial or extracranial sites observed an abscopal response in 11 patients (52%), including those with partial responses and with stable disease. The median time from radiotherapy to an abscopal response was 1 month (range, 1-4), and median overall survival was superior in patients exhibiting the abscopal effect compared with nonresponders (22.4 *vs* 8.3 months, respectively) [101, 107].

Another series included 47 metastatic melanoma patients who underwent radiotherapy following ipilimumab. A reduction of lesions was observed in 7 patients (11%) before radiation therapy compared with 16 (25%) after radiation therapy; in 11 of the latter (69%), an increase of lesions had been observed before radiotherapy (*P* = 0.03). The radiation fraction size < = 3 Gy was associated with favorable lesion response (*P* = 0.014) [101–108].

Chandra and collegues demonstrated no association between abscopal responses and either timing of ipilimumab in relation to radiotherapy or duration from first dose of ipilimumab to initiation of radiotherapy [109]. A subset of patients may have more favorable out-of-field responses following treatment with radiation. Interestingly, it has been described that multiple fraction radiation regimens were associated with a more favorable response [109].

### Abscopal effect and anti-PD1

The PD-L1 is another putative regulator of immune responses to radiation therapy [100]. Despite preclinical studies on its role in extending PFS and amplifying the abscopal response of radiation treatment [100], clinical data regarding abscopal effects with anti-PD1 are weak [101].

Although low doses of radiation increase T-cell infiltration, they also upregulate or increase PD-L1 expression levels on tumor cells and might contribute to radioresistance in tumors. Ribeiro and colleagues demonstrated an estimated rate of abscopal effect of 25% (3/12 patients), evaluating only patients with metastatic melanoma that had disease progression on anti-PD1, with radiotherapy median total dose of 24 Gy (1-40 Gy), averagely given in 3 fractions (1-10 fractions) [101].

Results from retrospective analyses indicated that the modality of the radiation treatment is crucial for minimizing toxicity. On this regard, significant reductions in toxicity have indeed been achieved with the use of more sophisticated radiation planning and treatment modalities, such as protons [106]. Questions arise also about the optimal time to begin radiotherapy as well as the right sequencing scheme between immune-modulation and radiation therapy in order to obtain an abscopal response [101].

Finally, it is difficult to discriminate on how much of the tumor response is due to systemic therapy alone or to combination treatment [101].

### Real life setting: special populations

In the real life setting, the clinicians have to treat different types of patients that are not widely represented in clinical trials, where the treated populations are selected on the basis of inclusion criteria: examples are patients with ECOG Performance Status higher than 2, elderly, patients with comorbidity, especially autoimmune disease, patients with uncontrolled brain metastases.

Due to immunosenescence, it is has been hypothesized that older adults may benefit less from immunotherapy [114–115]. There is limited information about the efficacy and toxicity of CPI in older adults, mostly derived from subgroup analysis of larger clinical trials, and no substantial differences in efficacy or safety were reported in elderly patients [116, 117]. Regarding Ipilimumab, no difference in median OS was found by Sileni et al. between patients aged ≥70 years (8.9 months (95% CI 7.2–10.6)) and < 70 years (7.0 months (95% CI 6.1–7.9); *P* = 0.17 with similar rates of immune related adverse events (irAE) [118]. Regarding AntiPD1, In CheckMate 066, in adults aged 65–75 and in >75 years aged treatment with nivolumab was associated with a HR of 0.44 (0.24–0.81) and a HR of 0.25 (0.10–0.61) respectively [11]. In CheckMate 069 the objective response rate was 64% in patients younger than 65 years compared to 53% in those aged 65 and older [12].

Some queries were about treating with immunocheckpoints patients with autoimmune comorbidities, because of possible exacerbations of autoimmune disorders. Major data about this issue derive from a retrospective study that reviewed 119 patients with either preexisting autoimmune disease and/or a history of irAEs during prior treatment with ipilimumab treated with anti–PD-1 therapy for advanced melanoma (30% of patients had active symptoms and 38% were on immunosuppressive agents): 38% of patients experienced a flare of their autoimmune, most of wich were mild in intensity. Efficacy was similar to literature: the overall response rate was 33%. As expected, the response rate in patients on immunosuppressants at the time of enrollment was lower [119].

In most phase II clincal trial, patients with clinical active untreated brain metastasis were excluded, and only stable treated brain lesions were admitted. Some retrospective trials evaluated the efficacy of antiPD1 in this particular population. In a recent ASCO annual meeting, data from a Phase I/II study underline the activity of antiPD1 also in this population. More data are needed to validate these aspect.

## CONCLUSIONS

Immune checkpoint inhibitors improved survival of melanoma patients and several strategies have been developed towards the improvement of their efficacy and activity. If the combination of anti-PD-1 and anti CTLA-4 is a new efficient immunotherapy regimen, promising results derive from the associations of immunocheckpoint inhibitors and the different systemic agents. To this extent, a larger number of treatment strategies will be soon available for melanoma patients.

Tissue and circulating predictive factors are largely awaited to help physician in their clinical practice; this need will be even more crucial in the very next future in order to tailor a personalized strategy in every single patient.
